# Prevalence and risk factors of low bone mineral density in spondyloarthritis and prevalence of vertebral fractures

**DOI:** 10.1186/s12891-017-1718-7

**Published:** 2017-08-22

**Authors:** Sandrine Malochet-Guinamand, Bruno Pereira, Zuzana Tatar, Anne Tournadre, Anna Moltó, Maxime Dougados, Martin Soubrier

**Affiliations:** 1 Rheumatology Department, Clermont-Ferrand University Hospital, 58 Rue Montalembert, FR 63003 Clermont-Ferrand, France; 2 Biostatistics unit (Clinical Research Direction), University Hospital of Clermont-Ferrand (CHU), Clermont-Ferrand, France; 3 Rheumatology Department, Paris Descartes University, Cochin Hospital, AP-HP, Paris, France

**Keywords:** Bone density, Fractures, Ankylosing spondylitis

## Abstract

**Background:**

Investigate the prevalence and risk factors of low bone mineral density (BMD) in patients with axial spondyloarthritis as well as investigating the prevalence of vertebral fractures.

**Methods:**

Patients underwent BMD measurements with dual-energy X-ray absorptiometry (DXA) in the anterior-posterior lumbar spine, lateral spine and hip. We screened for vertebral fractures using vertebral fracture assessment, and then checked for syndesmophytes on the VFA images. Sociodemographic and clinical variables were collected.

**Results:**

A total of 89 patients (41,6% female) took part in the study with a mean age of 44 ± 14 years and disease duration 10.2 ± 10.6 years. According to World Health Organization (WHO) criteria, 48,3% of patients displayed osteopenia and 6,7% osteoporosis. In the subgroup of women who underwent measurement at all sites including the lateral spine, the prevalence of osteopenia was 39.3% in the anterior-posterior spine, 32.1% in the lateral spine, and 64.3% with all sites together. VFA led to the diagnosis of at least one vertebral fracture in 6.2% of patients. On VFA, syndesmophytes were found in 24.3% of patients. The variables associated in multivariate analyses with low BMD in different measurement sites were low body mass index (BMI), a high physician’s global assessment score, a high Bath Ankylosing Spondylitis Functional Index (BASFI) score and female gender.

**Conclusion:**

Our study found a high prevalence (around 50%) of low BMD in SpA. Conversely, the prevalence of osteoporosis (6.7% according to WHO criteria) and vertebral fractures (6.2%) was lower than generally reported in the literature. While lateral spine BMD measurement did little to improve the detection of osteopenia in women, the sample size was not large enough to enable us to draw definite conclusions.

## Background

Spondyloarthritis (SpA) is the second most common form of inflammatory arthritis after rheumatoid arthritis. The Assessment in SpondyloArthritis international Society (ASAS) classification criteria define axial and peripheral forms [1]. Although current treatment, particularly with the advent of biological therapies, usually makes it possible to manage the disease well, a certain number of comorbidities may affect functional prognosis and alter quality of life. For instance, with a prevalence of 13%, osteoporosis was the most common comorbidity found in SpA in the ASAS-COMOSPA cross-sectional international study [2]. Most of the studies also show that patients with axial SpA (axSpA) have a higher prevalence of osteoporosis than that expected in the general population [3].

The EULAR SpA imaging task force recommends screening for osteoporosis in SpA [4]. The standard technique for measuring bone mineral density (BMD) is dual energy X-ray absorptiometry (DXA). Measurements are usually taken in the femur and in the lumbar spine in the anterior-posterior projection. Spine BMD measurements in the anterior-posterior projection take in the entire vertebral body as well as the posterior arch. Because of this, any type of SpA-related spinal damage, in particular syndesmophytes and posterior arch damage, may influence the measurements by overestimating them, thereby challenging the interpretation of the exam. For this reason, in the advanced disease stages, it is suggested to perform DXA spine measurement in the lateral projection [4]. The newer-generation densitometry machines can perform lateral spine measurement while the patient is in a supine position. This technique proves to exhibit good precision [5]. However, there is little data on the use of lateral spine measurement regarding its relevance in routine practice for osteoporosis screening.

The prevalence of vertebral fractures has been shown to be increased in ankylosing spondylitis (AS) from the early stages onwards [6]. Such spinal fractures can easily go unnoticed, given that they are sometimes asymptomatic, or when spine pain is interpreted as a symptom pertaining to the AS. Vertebral fracture assessment (VFA) is a low-radiation technique that allows vertebral fractures to be screened using DXA [7]. This means that vertebral fractures can be checked for while bone mass is being assessed, which is particularly useful since BMD assessment may underestimate the risk of fractures in this patient population [8]. There is as yet little data on the use of this technique in SpA.

The aims of this study were to evaluate the prevalence and risk factors of low BMD in the anterior-posterior spine, lateral spine, and hip in axial SpA patients, as well as the prevalence of vertebral fractures by means of VFA.

## Methods

### Population studied

This is a monocentric observational, cross-sectional study in axial SpA patients who presented consecutively in our rheumatology department. Patients provided their written consent and were included if they met ASAS criteria for axial SpA according to the Rheumatologist and were more than 18 years of age. The study was approved by the local ethics committee “Ile de France III” (S.C 3004), and all patients provided their informed consent to participate in the study.

### Demographic, clinical and laboratory data was collected during the visit

Demographic and clinical data included: Age, sex, weight, body mass index (BMI), smoking status (Never, history but cessation of more than 3 years, history but cessation of less or equal than 3 years, current smoker), consumption of alcohol, age at diagnosis, time since diagnosis, number of swollen joints, number of painful joints, physician’s global assessment of disease activity (evaluation of the activity of the disease based on the information provided by the patient and the data collected), use of non-steroidal anti-inflammatory drugs (NSAIDs) (yes or no and if yes % of days with NSAIDS intake), current prednisone intake (mg/day) and estimated total intake of corticosteroids from the beginning of the disease (mg), history of spinal fracture, history of peripheral fracture, calcium supplementation (never, past, current), vitamin D supplementation (never, past, current), anti-osteoporotic treatment (never, past, current), and disease activity and severity scores from the self-administered questionnaires (Bath Ankylosing Spondylitis Disease Activity Index [BASDAI] and Bath Ankylosing Spondylitis Functional Index [BASFI]).

Laboratory data (i.e. sedimentation rate, C-reactive protein (CRP), and vitamin D levels) were also reported and also Sacroiliitis on plevic X rays (at least garde II bilateral or grade III unilateral: No; Yes; Non applicable or not done).

### BMD measurement

All participants in our center underwent DXA BMD measurement in the anterior-posterior spine (L1 to L4), lateral spine (L2 to L4) and left hip (total hip and femoral neck), or in the right hip if the examination was not feasible in the left hip, for instance if there had been hip replacement. All measurements were conducted by technicians trained on the Hologic Discovery system according to the manufacturer’s recommendations.

The results were expressed in BMD (BMD g/cm^2^) as well as in a Z- and T-score using the reference curves supplied by the manufacturer. The Z-score is the number of standard deviations above or below the values of healthy adults of the same age and same sex. The T-score is the number of standard deviations above or below the values of young adults of the same sex.

There is no agreement on what defines low BMD in young adults. The International Society for Clinical Densitometry recommends using a Z-score of ≤ −2 standard deviations as the threshold for low BMD in this population [9].

In postmenopausal women, and in men aged more than 50 years, the World Health Organization (WHO) defines osteopenia as a T-score of between −1 and −2.5 and osteoporosis as a T-score of ≤ − 2.5 [10]. Our study population was not large enough to apply both types of definition on the basis of age and menopausal status. We therefore extrapolated and used the WHO definitions for the entire study population.

For lateral spine BMD measurement, reference curves, and therefore Z- and T-score data, are only available for women. Definitions of low bone mass and osteoporosis are the same as at other sites.

Vertebral fractures were checked for by means of VFA using the software supplied by the manufacturer. T4 to L4 were qualitatively analyzed and then semi-quantitatively analyzed using the Genant classification [11]. Quantitative analysis was then performed using the vertebral height measurement software supplied by the manufacturer. A physician trained in reading VFA read the VFA images and detected vertebrae deemed to be fractured. The height of vertebrae deemed to be fractured was then measured using the morphometry software supplied by the manufacturer. These vertebrae were then categorized according to severity by means of the Genant classification. Grade 1 (mild) was defined as a decrease in vertebral height (anterior, middle or posterior) of 20 to 25%; Grade 2 (moderate) as a decrease in vertebral height of 25 to 40%; and Grade 3 (severe) as a decrease in vertebral height of more than 40%.

Additionally, as described by Aubry-Rozier in a pilot study, each intervertebral disk space from T12-L1 to L5-S1 was studied so as to check whether any syndesmophytes were present. In this study overall agreement beetween VFA and X-rays was excellent [12].

The machine was checked by the daily measurement of a phantom supplied by the manufacturer and sent to an authorized quality-control body.

### Statistical analysis

All analyses were performed using Stata software (Version 13, StataCorp, College Station, TX) and were conducted for a two-sided Type I error of 5%. Continuous data were presented as mean ± standard deviation or median [interquartile range], and categorical parameters as the number of patients and associated percentages. To highlight the risk factors of low bone mineral density and of vertebral fracture assessment (in other words, in order to study the intensity of AP lumbar spine, femoral neck, total hip and lateral lumbar spine compared to patients’ characteristics), analyses have considered usual statistical tests: [1] ANOVA or Kruskal-Wallis tests if the conditions for ANOVA were not met ((i) normality and (ii) homoscedasticity analyzed using Bartlett’s test for the comparisons between gender, smoking status and Vitamin D supplementation followed when appropriate (omnibus *p*-value < 0.05) by post-hoc tests to take into account multiple comparisons (Tukey-Kramer’s test after ANOVA and Dunn’s test after Kruskal-Wallis), [2] correlation coefficients (Pearson or Spearman according to statistical distribution), applying Sidak’s correction of type I error, for the study of relationships with other quantitative variables (age, BMI, disease duration, number of swollen joints, number of tender joints, ESR, CRP, physician’s global assessment, percentage of days with NSAID intake, current vitamin D level, BASFI and BASDAI). When necessary, comparisons between categorical data were carried out by the Chi-squared test or, when appropriate, by Fisher’s exact test. Then, multivariate analyses were performed to study the parameters associated to low bone mineral density and vertebral fracture assessment. More precisely, multiple linear regression models were performed using the stepwise approach (backward and forward) considering covariates selected according to univariate results and clinical relevance. The normality of residuals was studied as described previously and the results were expressed as regression coefficients and 95% confidence intervals.

## Results

In total, 89 patients meeting the criteria for axial SpA participated in the study. There were 37 women (41.6%) and 52 men (58.4%). Their mean age was 44 years (±14) and they had a mean disease duration of 10.2 years (±10.6). 55 (62%) patients had sacroilitis on pelvic X-rays (at least grade II bilateral or grade III unilateral).

The complete characteristics of the study population are summarized in Table 1.

**Table 1:** Population characteristics

Variable	Mean ± SD
Age, years	44.4 ± 14
Sex, number (%)	
Male	52 (58.4)
Female	37 (41.6)
Disease duration (years)	10.2 ± 10.6
BMI (kg/m^2^)	26 ± 4.2
ESR (mm 1st H)	10 ± 9.5
CRP (mg/dL)	7.1 ± 11.8
Physician’s global assessment (0–10)	4.2 ± 2.6
Percentage of days with NSAID intake (%)	64 ± 31.3
Current vitamin D level (ng/mL)	22.6 ± 9.4
BASFI score (0–100)	27.1 ± 23.5
BASDAI score (0–10)	6.1 ± 4.8
Femoral neck BMD (g/cm^2^)	0.795 ± 0.116
Total hip BMD (g/cm^2^)	0.926 ± 0.121
AP lumbar spine BMD (g/cm^2^)	1.017 ± 0.172
Lateral lumbar spine BMD (g/cm^2^)	0.926 ± 0.121

*BMI* body mass index, *ESR* erythrocyte sedimentation rate, *CRP* C-reactive protein, *BASFI* bath ankylosing spondylitis functional index, *BASDAI* bath ankylosing spondylitis disease activity index, *BMD* bone mineral density

When WHO definitions were applied to the entire population, 48.3% of patients exhibited osteopenia and 6.7% osteoporosis. When comparing the different sites, the femoral neck was the measurement site that most often enabled a diagnosis of osteopenia to be made (42.7%, compared to 25.8% in the total hip and 31.8% in the anterior-posterior spine; *p* = 0.003). Five patients (5.6%) had a Z-score ≤ −2.

Overall, 28 of the 37 women underwent spine BMD measurement in the anterior-posterior projection and lateral projection (the measurement was not performed at this site or was uninterpretable in 9). In total, 32.1% displayed osteopenia on lateral spine measurement and 39.3% osteopenia on anterior-posterior spine measurement. Osteopenia at at least one site was found in 57% of these 28 women when the anterior-posterior spine and hip were taken into account. Lateral spine measurement detected two additional osteopenic patients with normal BMD in the hip and anterior-posterior spine (Table 2). The overall prevalence of osteopenia in this group was 64.3%. In addition, the entire population displayed significantly lower spine BMD on lateral measurement than on anterior-posterior measurement (0.809 ± 0.134 vs. 1 ± 0.136; *p* < 0.001). Women had lower BMD than men on total hip measurement (0.89 ± 0.11 vs. 0.95 ± 0.12; *p* = 0.04).

**Table 2:** Number of osteopenic female patients when considering the lateral lumbar spine and other measurement sites

	Lateral lumbar spine T score ≥ −1 SD, *n*	Osteopenia at lateral lumbar spine, *n*	Total
T score ≥ −1 SD in all other measurement sites, *n*	10	2	12
Osteopenia at at least one other measurement site, *n*	9	7	16
Total	19	9	28

### Vertebral fractures

VFA analysis of the lateral spine was possible in 80 patients (data not available for four and non-analyzable for five). A fracture was detected in five patients (6.25%), with a total of eight fractures detected. 2 fractures were detected in the 34 patients without sacroilitis (5,9%) and 3 in the 55 patients with sacroilitis (5,4%).

### Syndesmophytes

Screening for syndesmophytes was possible in 78 patients, with syndesmophyte observed at at least one level in 19 of them (24.3%). The most frequently involved level was T12/L1 (12 patients), with a mean of 1.7 levels affected.

Regardless of the site, no significant difference was found with respect to BMD or T-score when considering the two groups of patients according to whether they had or had not syndesmophytes. No significant association between BMD, regardless of the site, and the number of levels with syndesmophytes was observed.

### Factors associated with BMD according to site

The association between BMD and the different risk factors has been summarized in Table 3 (expressed as correlation coefficient) and Table 4 (mean ± SD and *p* value for statistical tests.)

**Table 3:** Variables correlation with BMD in differents measurement sites using univariated analysis

	AP LS	*p*	Femoral neck	*p*	Total hip	p	Lateral LS	*p*
Age	*0.11* ^*a*^	0.31	*−0.22* ^*a*^	0.03	*−0.003* ^*a*^	0.97	*−0.08* ^*a*^	0.52
BMI	*0.27* ^*a*^	0.01	*0.24* ^*a*^	0.02	*0.33* ^*a*^	0.002	0.05^*a*^	0.62
Disease duration	*0.02* ^*a*^	0.86	*−0.17* ^*a*^	0.13	*−0.13* ^*a*^	0.21	*−0.02* ^*a*^	0.84
Number of swollen joints	−*0.20* ^*a*^	0.06	*−0.005* ^*a*^	0.96	*−0.10* ^*a*^	0.34	*−0.25* ^*a*^	0.03
Number of tender joints	*−0.22* ^*a*^	0.04	*−0.08* ^*a*^	0.47	*−0.12* ^*a*^	0.25	*−0.32* ^*a*^	0.005
ESR	*−0.0008* ^*a*^	0.99	*−0.08* ^*a*^	0.44	*−0.09* ^*a*^	0.37	*0.02* ^*a*^	0.87
CRP	*0.12* ^*a*^	0.26	*−0.05* ^*a*^	0.63	*0.05* ^*a*^	0.67	*0.13* ^*a*^	0.28
Physician’s global assessement	*−0.24* ^*a*^	0.03	*−0.20* ^*a*^	0.06	*−0.21* ^*a*^	0.05	*−0.25* ^*a*^	0.04
% of days with NSAID intake	*−0.003* ^*a*^	0.97	*−0.16* ^*a*^	0.15	*−0.11* ^*a*^	0.31	*0.07* ^*a*^	0.57
Current vit D level	*−0.15* ^*a*^	0.16	*−0.18* ^*a*^	0.09	*−0.23* ^*a*^	0.03	*−0.07* ^*a*^	0.56
BASFI	*−0.10* ^*a*^	0.37	*−0.22* ^*a*^	0.04	*−0.19* ^*a*^	0.07	*−0.10* ^*a*^	0.41
BASDAI	*−0.26* ^*a*^	0.01	*−0.17* ^*a*^	0.10	*−0.19* ^*a*^	0.06	*−0.29* ^*a*^	0.01

^a^:correlation coefficient [95% CI]; p, *p* value. BMD, Bone mineral density; AP, anterior-posterior; LS, lumbar spine; BMI, body mass index; ESR, erythrocyte sedimentation rate; CRP, C-reactive protein; BASFI, bath ankylosing spondylitis functional index; BASDAI, bath ankylosing spondylitis disease activity index

**Table 4:** BMD comparaison between groups for qualitatives variables

	AP LS		Femoral neck		Total hip		Lateral LS	
	BMD g/cm^2^ Mean ± SD	*p*	BMD g/cm^2^ Mean ± SD	*p*	BMD g/cm^2^ Mean ± SD	*p*	BMD g/cm^2^ Mean ± SD	*p*
Gender								
Female	0.984 ± 0.135		0.778 ± 0.114		0.894 ± 0.113		0.79 ± 0.11	
		0.24		0.25		0.04		0.17
Male	1.04 ± 0.19		0.807 ± 0.117		0.948 ± 0.122		0.82 ± 0.14	
Smoking status								
Never	0.980 ± 0.131		0.794 ± 0.121		0.934 ± 0.120		0.808 ± 0.141	
ces >3 years	1.088 ± 0.265	0.47	0.797 ± 0.102	0.98	0.934 ± 0.109	0.78	0.831 ± 0.154	0.54
ces ≤3 years	1.048 ± 0.578		0.804 ± 0.089		0.888 ± 0.098		0.892 ± 0.074	
Current	1.016 ± 0.138		0.796 ± 0.127		0.921 ± 0.134		0.790 ± 0.120	
Vitamin D suppl								
Never, n (%)	1.015 ± 0.15		0.805 ± 0.093		0.927 ± 0.103		0.814 ± 0.128	
Past, n (%)	1.016 ± 0.12		0.783 ± 0.138		0.921 ± 0.140		0.814 ± 0.148	
Current, n (%)	1.029 ± 0.275	0.66	0.794 ± 0.130	0.58	0.927 ± 0.129	0.98	0.811 ± 0.141	0.93

*BMD* bone mineral density, *AP* anterior-posterior, *LS* lumbar spine, *ces* cessation, *suppl* supplementation, *p p* value

The variables that significantly correlated with low BMD were in the anterior-posterior spine, a low BMI, the number of painful joints, a high physician’s global assessment score and a high BASDAI score; in the femoral neck, the variables correlated with low BMD were age, a low BMI, and a high BASFI score; in the total hip, these variables were low BMI, a high physician’s global assessment score, and high vitamin D levels. Lastly, in the lateral spine, the number of painful joints, number of swollen joints, high physician’s global assessment score, and high BASDAI score all correlated with low BMD.

Regardless of the site, there was no significant association between BMD and smoking status, the length of time the patient had suffered from the disease, laboratory signs of inflammation (sedimentation rate and CRP), the percentage of days taking NSAIDs, and vitamin D supplementation.

The variables that were associated with BMD at the different sites were analyzed using multiple linear regression. The results have been summarized in Table 5. The following were significantly associated with low BMD: in the anterior-posterior spine, low BMI and a high physician’s global assessment score; in the femoral neck, low BMI and a high BASFI score; and in the total hip, low BMI, female sex, and the physician’s global assessment score. No significant association was found with the lateral spine.

**Table 5:** Results from multiple linear regression analysis with BMD at different measuring sites

	AP lumbar spine BMD^a^	Femoral Neck BMD^a^	Total hip BMD^a^
BMI	0.01 [0.01; 0.02]	0.01 [0.01; 0.02]	0.01 [0.01; 0.02]
Gender			
(Male/female)			0.04 [0.01; 0.09]
Physician’s global assessement	−0.01 [−0.03; −0.01]		−0.01 [−0.02; 0.0]
BASFI		−0.01 [−0.02; −0.01]	

^a^Regression-coefficient [95%IC]. *BMD* bone mineral density, *BMI* body mass index, *BASFI* bath ankylosing spondylitis functional index

## Discussion

Our study showed that 48% of patients being followed for axial SpA displayed osteopenia and 6.7% osteoporosis according to WHO criteria. These results reveal a relatively low prevalence of osteoporosis in our population compared with the data published in the scientific literature. For instance, in a 2012 literature review, the prevalence of osteopenia according to the WHO definition was 54% in the spine and 51% in the hip, whereas that of osteoporosis at the same sites was 13 and 16% respectively [13].

The difference in the prevalence of osteoporosis observed in our study may be linked to the different characteristics of our study population. Our study included SpA patients as defined by the ASAS criteria, whereas the above-mentioned review included patients who were suffering from AS according to the modified New York criteria. In a more recent review involving 55 studies, the prevalence of osteopenia in patients with AS or SpA varied between 5 and 88%, whereas that of osteoporosis ranged from 3 to 47% [3].

BMD was lower in the lateral spine than in the anterior-posterior spine. However, using lateral spine measurement did little to improve the detection of low BMD in our study since it led to an osteopenia diagnosis in only additional two female patients who had not met criteria for osteopenia in the other measurement sites. Nor was lateral spine measurement more effective in our study than anterior-posterior spine measurement for screening for low BMD. This contrasts with MA Ulu et al. in patients with AS meeting the modified New York criteria in patients who had had the disease for more than 10 years, they showed that lateral and not anterior-posterior measurement was lower in patients than in controls [14]. In a study in AS patients lateral spine measurement led to more diagnoses of osteoporosis than did anterior-posterior spine measurement [15]. Nevertheless, these two studies do not answer the question of whether lateral spine measurement improves the prediction of osteoporosis compared with the combined use of hip and anterior-posterior spine measurements. For example, in two other studies using lateral spine measurement, hip measurement was just as discriminative, being lower in patients than in controls [14, 16].

The prevalence of vertebral fractures in our study was 6.25%, a figure mostly lower than those reported in the literature. That said, the prevalence of vertebral fractures in SpA has been shown to vary greatly depending on the studies. A literature review found a prevalence of vertebral fracture in AS that varied between 4 and 41% [17]. Vertebral fractures are often defined in studies as a reduction in vertebral height relative to the other vertebrae. But this takes no account of the deformities’ etiology. Hence some deformities associated with the disease, may be deemed to be fractures if a morphometric technique is used without the images being read by an expert, and so lead to an overestimation of fractures. More recently, the VFA technique has been validated for screening for vertebral fractures, showing good agreement with semiquantitative reading of standard radiographs and excellent negative predictive value [7]. One study in AS patients showed on average, good agreement between VFA using DXA and radiographic interpretation [18]. Geusens et al. investigated the prevalence of vertebral fractures in SpA patients by means of the semiquantitative interpretation of standard radiographs and VFA. They employed the Genant method, and found an 11.8% prevalence of vertebral fractures on the radiographs along with good agreement between the two assessment modalities [19]. In a population with early SpA, 15% of patients had already suffered from at least one fracture as assessed by standard radiography [20]. However, the authors pointed out that the method they used for interpreting the radiographs did not enable them to distinguish between the causes of vertebral height reduction.

Lastly, in our study population, 23.4% of patients displayed syndesmophytes at at least one vertebral level on VFA analysis. We found no association between the presence of syndesmophytes and bone density or T-score regardless of the site. In contrast, MA Ulu showed that AS patients with at least one syndesmophyte on standard radiography had lower BMD than the others in the lateral spine projection [14]. The modified Stoke Ankylosing Spondylitis Spine Score, or mSASSS, which evaluates AS-related radiographic lesions, is also a factor that may be associated with low BMD, particularly in the femoral neck or spine on volumetric analysis but not in the anterior-posterior projection [15]. This mSASSS may even be associated with increased BMD in the spine [21]. The lack of association between syndesmophytes and BMD in our study may be linked to the fact that most cases displayed moderate involvement. It is different from the results published by Wildberger et al. In this study men with syndesmophytes have a normal BMD spine T-score [22]. Furthermore, this assessment modality has not been sufficiently validated as yet and requires additional study.

In our study, BMD was lower in women than men in the total hip only. Depending on the site, the variables significantly associated with lower BMD were, on univariate analysis, age, female sex, lower BMI, number of painful joints, number of swollen joints, physician’s global assessment, BASDAI, BASFI, and vitamin D levels; on multivariate analysis, they were sex, BMI, physician’s global assessment and BASFI. These results are consistent with some of the data in the literature. Factors that have been described as being associated with BMD in SpA or AS include demographic variables, disease duration, and disease activity or severity. Eva Klingberg studied the prevalence of osteoporosis in AS by means of DXA including lateral spine measurements [14]. Low BMD was associated with age, disease duration, low BMI, inflammatory parameters, and structural parameters such as the mSASSS or a spinal mobility index like the Bath Ankylosing Spondylitis Metrology Index (BASMI). Disease monitoring indexes were not associated with low BMD. Cross-sectional studies have returned controversial results on the correlation between BMD, disease activity and inflammatory parameters [3] while some prospective studies have shown that bone loss is greater in the active forms of the disease [23, 24]. Signs of inflammation on magnetic resonance imaging are associated with lower BMD in patients with inflammatory back pain and symptoms suggestive of SpA [25] or in patients with early non-radiographic SpA [26].

The variable that was the most consistently and most strongly associated with low BMD in our study was low BMI, which is known to be a major risk factor for osteoporosis and fracture in the major international cohorts [27]. We also found that BMD at the femoral neck was inversely correlated with BASFI. Other studies have shown a high BASFI score to be a risk factor for low BMD [14, 16, 21, 28] but this has not been so in all studies [15, 25, 29]. A loss of mobility is also a classic risk factor for bone loss [30]. It can be assumed that disease-related changes in functional capacity may be one of the causes of bone loss in SpA.

However, we found no association in our study with certain parameters such as smoking, CRP, use of NSAIDs, and vitamin D supplementation. Although there is little data available on the use of NSAIDs, in the prospective study of the DESIR cohort of patients with recent inflammatory back pain, the use of NSAIDs was shown to prevent bone loss in the hip [31]. We didn’t take into account post-menopausal status that was not available and could interfer with women bone density.

The utility of our study is that it investigated DXA BMD in patients with axial SpA regardless of disease severity or duration, that it included lateral spine measurement, and that VFA was performed in all cases, enabling the detection of fractures and analysis of syndesmophytes.

That said, our study displays several limitations. The relative small sample size limits the conclusions that can be drawn, specially associated factors and also prevalence of osteoporosis and fracture. For instance, a criticism can be made of our applying the WHO definitions of osteopenia and osteoporosis to the entire study population. Yet, we lacked the power to investigate both types of population separately, and very few patients (5.6%) met the definition of low BMD based on a Z-score ≤ −2.

## Conclusion

Our study found a high prevalence (around 50%) of low BMD in SpA in our center. Conversely, the prevalence of osteoporosis (6.7% according to WHO criteria) and vertebral fractures (6.25%) was lower than those usually reported in the scientific literature.

It appears essential to assess the standard risk factors for osteoporosis in this population.

Lateral spine BMD measurement did little to improve the detection of osteopenia in women. We need new toll to improve bone fragility detection in this population. TBS (Trabecular Bone Score) measures the bone texture, and could predict fracture risk independently of BMD in secondary causes of osteoporosis [32] and could be helpfull in AS [22]. It may also be worthwhile further defining which deformities should be taken as vertebral fractures in this patient population.

## Abbreviations

AS: Ankylosing spondylitis
ASAS: SpondyloArthritis international Society
axSpA: axial SpA
BASDAI: Bath ankylosing spondylitis disease activity index
BASFI: Bath Ankylosing spondylitis functional index
BMD: Bone mineral density
BMI: Body mass index
CRP: C-reactive protein
DXA: Dual energy X-ray absorptiometry
NSAIDs: Non-steroidal anti-inflammatory drugs
SpA: Spondyloarthritis
VFA: Vertebral fracture assessment
WHO: World Health Organization
